# Vanadia supported on nickel manganese oxide nanocatalysts for the catalytic oxidation of aromatic alcohols

**DOI:** 10.1186/s11671-015-0750-5

**Published:** 2015-02-06

**Authors:** Syed F Adil, Saad Alabbad, Mufsir Kuniyil, Mujeeb Khan, Abdulrahman Alwarthan, Nils Mohri, Wolfgang Tremel, Muhammad Nawaz Tahir, Mohammed Rafiq Hussain Siddiqui

**Affiliations:** Department of Chemistry, College of Science, King Saud University, P.O. 2455, Riyadh, 11451 Kingdom of Saudi Arabia; Institute for Inorganic and Analytical Chemistry, University of Mainz, Duesbergweg 10-14, 55128 Mainz, Germany

**Keywords:** Catalysis, Vanadia nanoparticles, Mixed metal oxide

## Abstract

Vanadia nanoparticles supported on nickel manganese mixed oxides were synthesized by co-precipitation method. The catalytic properties of these materials were investigated for the oxidation of benzyl alcohol using molecular oxygen as oxidant. It was observed that the calcination temperature and the size of particles play an important role in the catalytic process. The catalyst was evaluated for its oxidation property against aliphatic and aromatic alcohols, which was found to display selectivity towards aromatic alcohols. The samples were characterized by employing scanning electron microscopy, transmission electron microscopy, X-ray diffraction, Brunauer-Emmett-Teller analysis, thermogravimetric analysis, and X-ray photoelectron spectroscopy.

## Background

Catalysis, which is largely a surface phenomenon, is an area of research that has been a widely studied subject by scientists and technologists [1-4]. However, the zeal for finding a better performing catalyst for various processes including CO oxidation [5], Fischer-Tropsch synthesis [6], MOFs for biomimetic catalysis [7], fuel cell reactions [8], and selective hydrogenolysis of aryl ethers [9] still is an ongoing process. Among several elements that are being tested and tried for catalysis, vanadium oxide and other compounds containing vanadium have attracted significant attention as catalyst for many oxidation reactions [10-14]. Apart from this, vanadium oxide has also been explored for various other applications including pseudocapacitors [15] and cathode material [16] in various conversion reactions of alkanes to alkenes, organic acids, and the synthesis of light olefins by means of oxidative dehydrogenation (ODH) [17-22]. Furthermore, the catalytic oxidation properties of vanadium-based catalysts have also been extensively exploited for several other reactions such as conversion of propane to CO_x_/H_2_ [23], propane partial oxidation [24], oxidation of SO_2_ [25], formaldehyde to formic acid [26], oxidation of α-hydroxy ketones, α-hydroxy esters [27], aerobic oxidative cleavage of secondary-tertiary glycols [28], and oxidative dehydrogenation of ethane [29]. In some cases, it has also been used as support material for other catalysts, e.g., Pt nanoparticles, supported by vanadia-decorated carbon nanotubes for methanol electro-oxidation reaction [30]. Notably, vanadium has displayed excellent catalytic activities in all forms, whether it has been employed as a supported active phase or in the form of mixed oxides prepared in combination with other ions; it displayed efficient catalytic properties as an oxidation catalyst.

Recently, mixed metal oxides (MMO) have attracted significant attention as solid catalysts, due to their low cost, easy regeneration, selective action, and excellent acid–base redox properties [31]. Among various MMO, manganese-based MMO have attracted much attention due to their higher catalytic performances [32]. Several catalytic reactions using manganese-oxide-based MMO have been reported. Examples include the catalytic reaction of hydrogen production via autothermal reforming of ethanol [33], steam reforming of tar from biomass pyrolysis [34], methane combustion at low temperature [35], and enhanced glucose electrooxidation [36] carried out using nickel manganese MMO. Our group has been involved in the synthesis of various MMOs [37] and evaluated their catalytic performance for several organic transformations [38]. In this study, to exploit the excellent catalytic activity of vanadium oxide, we report the synthesis of heterogeneous catalysts based on vanadium oxide nanoparticles supported on nickel manganese oxide MMO. The as-prepared catalysts were characterized using various spectroscopic and microscopic techniques including transmission electron microscopy (TEM), scanning electron microscopy (SEM), X-ray diffraction (XRD), X-ray photoemission spectroscopy (XPS), and thermogravimetric analysis (TGA), and their catalytic activities were evaluated for the oxidation of various aromatic alcohols.

## Methods

### Preparation of vanadium oxide supported on nickel manganese oxide by deposition method

Ninety-five milliliters of 0.2 M solutions of nickel nitrate and manganese nitrate were mixed in a round-bottomed flask, followed by addition of 10 mL of 0.2 M solution of vanadium chloride. The resulting solution was heated to 80°C under stirring using a mechanical stirrer. A 1 M solution of NaHCO_3_ was added dropwise until the solution attained a pH 9. The solution was continuously stirred at the same temperature for about 3 h and left on stirring over night at room temperature. The solution was filtered using a Buchner funnel under vacuum and then dried at 70°C overnight. The product obtained was characterized using SEM, TEM, EDAX, XRD, XPS, Brunauer-Emmett-Teller (BET), and TGA. The resulting powder was then calcined at different temperatures and evaluated for its oxidation activity for the oxidation of benzyl alcohol as a model precursor.

### Catalyst testing

In a typical reaction, 300 mg of catalyst was loaded in a glass flask pre-charged with 0.2 mL (2 mmol) benzyl alcohol mixed with 10 mL toluene as a solvent; the mixture was then refluxed at 100°C, and oxygen was bubbled at a flow rate of 20 mL min^−1^ into the mixture under vigorous stirring. After reaction, the solid catalyst was separated by centrifugation, and the liquid samples were analyzed by gas chromatography to evaluate the conversion of the desired product using an Agilent 7890A GC (Agilent Technologies, Inc., Santa Clara, CA, USA), equipped with a flame ionization detector (FID) and a 19019S-001 HP-PONA column.

### Catalyst characterization

SEM and elemental analysis (energy-dispersive X-ray analysis (EDX)) were carried out using a Jeol SEM model JSM 6360A (JEOL Ltd., Akishima-shi, Japan). This was used to determine the morphology of nanoparticles and its elemental composition. TEM was carried out using a Jeol TEM model JEM-1101 (JEOL Ltd., Akishima-shi, Japan), which was used to determine the shape and size of nanoparticles. Powder X-ray diffraction studies were carried out using an Altima IV (Make: Rigaku, Shibuya-ku, Japan) X-ray diffractometer. Fourier transform infrared spectroscopy (FT-IR) spectra were recorded as KBr pellets using a PerkinElmer 1000 FT-IR spectrophotometer (PerkinElmer, Waltham, MA, USA). BET surface area was measured on a NOVA 4200e surface area and pore size analyzer (Quantachrome Instruments, FL, USA). Thermogravimetric analysis was carried out using PerkinElmer Thermogravimetric Analyzer 7 (PerkinElmer, Waltham, MA, USA). XPS was measured on a PHI 5600 Multi-Technique XPS (Physical Electronics, Lake Drive East, Chanhassen, MN, USA) using monochromatized Al Kα at 1486.6 eV. Peak fitting was performed with CASA XPS Version 2.3.14 software.

## Results and discussion

### Catalyst characterization

**Figure 1:**
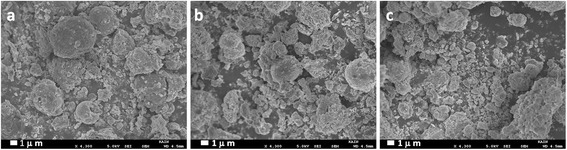
**SEM of the catalyst (a) V**_2_ **O**_5_ **(1%)-NiMnO, (b) V**_2_ **O**_5_ **(3%)-NiMnO, and (c) V**_2_ **O**_5_ **(5%)-NiMnO at 300°C.**

The morphology and the particle size of the synthesized catalyst were characterized using SEM and TEM. The SEM micrographs of the pre-calcined (300°C) catalyst V_2_O_5_ (X%)-NiMnO, where *X* = (1, 3, and 5), are shown in Figure 1. It was observed that the morphology of the synthesized catalysts is not well defined, and the surface appears to be rugged without any obvious phase separation. The stoichiometric amount of elements was confirmed from the EDX analysis and found to be approximately in agreement with the calculated value.

**Figure 2:**
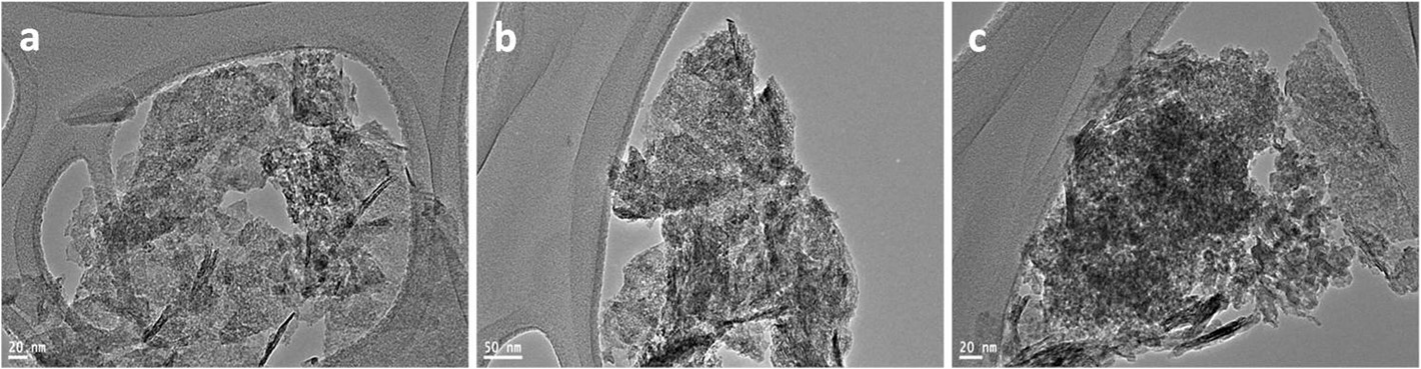
**TEM of the catalyst (a) V**_2_ **O**_5_ **(1%)-NiMnO, (b) V**_2_ **O**_5_ **(3%)-NiMnO, and (c) V**_2_ **O**_5_ **(5%)-NiMnO at 300°C.**

The TEM image of the catalyst V_2_O_5_ (X%)-NiMnO (*X* = 1, 3, 5) was carried out to investigate the shape and size of the particles more closely (Figure 2). The average particle size was calculated using image-processing program Image J software of the image adjacent to it. It was found that the synthesized catalyst 1% and 3% V_2_O_5_-NiMnO possessed particle sizes of 2.8 and 2.7 nm, respectively, whereas V_2_O_5_ (5%)-NiMnO was found to contain particles of size 2.2 nm. The TEM image analysis of the other synthesized catalysts gave information of the particles to be around 3 to 4 nm in size.

### XRD spectrum

**Figure 3:**
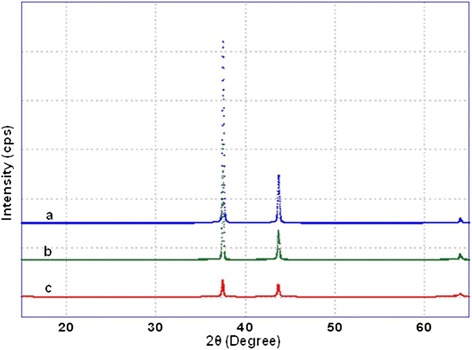
**XRD pattern of catalyst (a) V**_2_ **O**_5_ **(1%)-NiMnO, (b) V**_2_ **O**_5_ **(3%)-NiMnO, and (c) V**_2_ **O**_5_ **(5%)-NiMnO calcined 300°C.**

Figure 3 shows X-ray diffraction patterns of mixed oxides of nickel manganese with different % of vanadium oxide nanoparticles pre-calcined at 300°C. The structural results are listed in Table 1. The analysis of the XRD spectrum of X% V_2_O_5_-NiMnO (Figure 3), where *X* = (1, 3, and 5) of pre-calcined at 300°C showed that the V_2_O_5_ (1%)-NiMnO, V_2_O_5_ (3%)-NiMnO, and V_2_O_5_ (5%)-NiMnO contain reflections corresponding to cubic hexanickel manganese(IV) oxide (ICSD # 40584). No reflection corresponding to vanadium oxide was observed which could be due to the low percentage present in the catalyst.

**Table 1:** **Textural and structural properties of vanadium-oxide-doped nickel manganese oxide**

**Sample**	**Loading [wt%]**	**Calcination temperature (°C)**	***S***_BET_	***D*** **[mm]**	**Phase**
1V_2_O_5_	1	300	68.28	14.477	Ni_6_MnO_4_
3V_2_O_5_	3	300	62.81	12.269	Ni_6_MnO_4_
5 V_2_O_5_	5	300	98.54	20.804	Ni_6_MnO_4_
5V_2_O_5_	5	400	57.00	16.908	Ni_6_MnO_4_
5V_2_O_5_	5	500	20.97	12.326	Ni_6_MnO_4_

*S*_BET_ is the specific surface area. *D* is the crystal domain size calculated by using Scherrer Equation. Phase detected by XRD.

### XPS analysis

**Figure 4:**
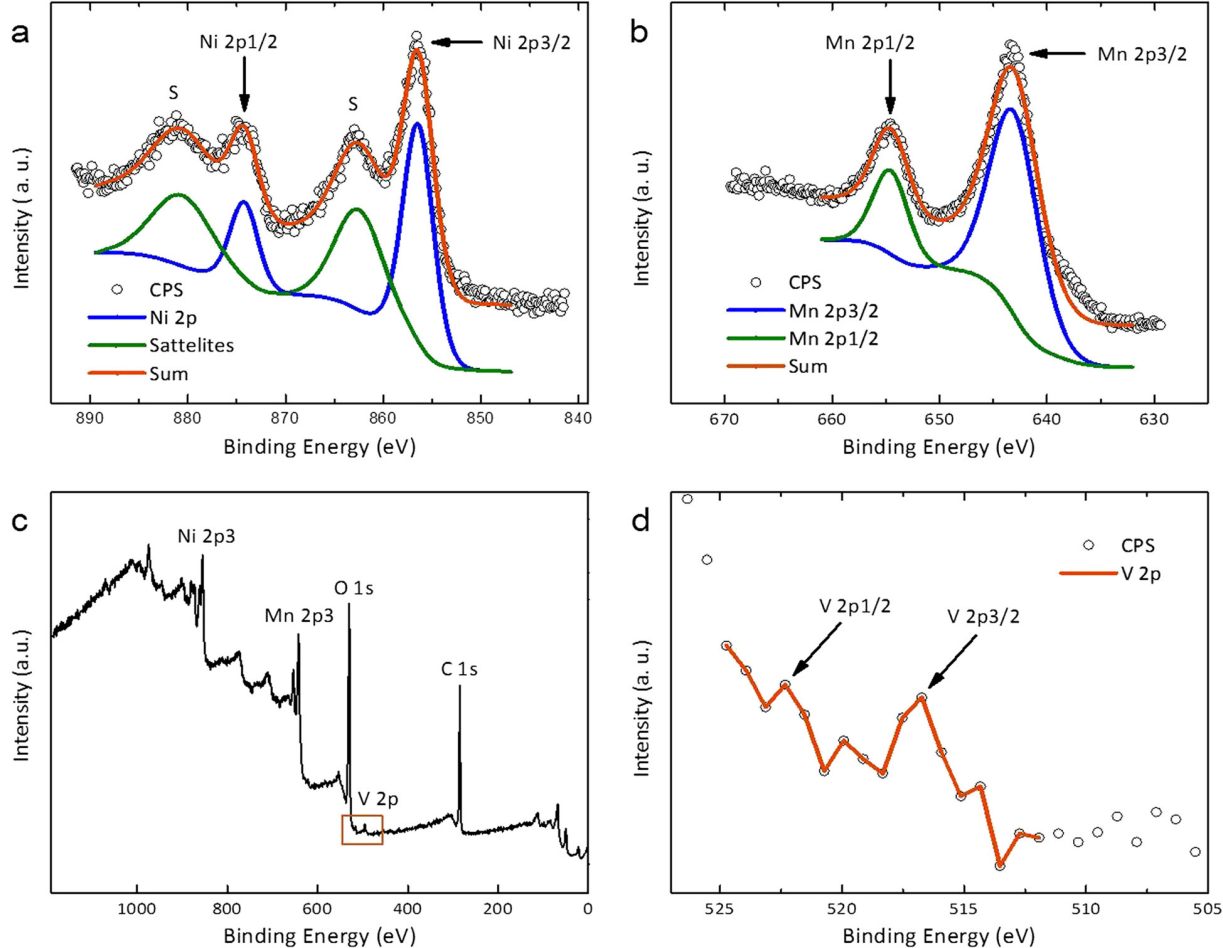
**XPS-spectra of V**_2_ **O**_5_ **(5%)** **-NiMnO. (a)** Ni 2p spectrum. Blue: Fit for Ni 2p_3/2_- and 2p_1/2_-peak. Yellow: Fit for the two satellite peaks. Red: Envelope of both fits. **(b)** Mn 2p spectrum. Blue: Fit for Mn 2p_3/2_-peak. Yellow: Fit for Mn 2p_1/2_-peak. Red: Envelope of both fits. All fits were shifted to lower intensity for better visibility. **(c)** Spectrum over whole binding energy range. Ni, Mn, V, C, and O are marked at the highest intensity peaks. **(d)** Magnification of V 2p peak as marked in **(c)** showing the position of the 2p_1/2_- and 2p_3/2_-peak.

The distinct amount of vanadium oxide supported on the surface region and the oxidation state of the vanadium were confirmed using XPS studies. The spectrum is given in Figure 4. It was also intended to establish the phase changes if any on the surface of the catalytic system before and after catalyzing the oxidation reaction. It was observed that there is no significant change in the spectrum obtained for the catalysts before and after reaction. Two weak signals observed at a binding energy (BE) of 517.0 and 522.4 eV indicate that the oxidation state of +5 for vanadium is present in the catalyst, which agree well with the results published by Silversmit et al. [39]. The very low percentage amount of vanadium could be responsible for the weak signal. The binding energies obtained for manganese and nickel (see Table 2) suggest that the oxidation states are +4 and +2, respectively [40,41], which corroborates the results obtained from the XRD. There was no change observed in the binding energies corresponding to the manganese and nickel after the reaction indicating that there is no change in oxidation state of the metals.

**Table 2:** **Binding energies of transition metal compounds calculated from the maxima in the XPS spectra**

**Compound**	**BE**_1_ **(2p**_1/2_ **) (eV)**	**BE**_2_ **(2p**_3/2_ **) (eV)**	Δ**E (BE**_1_ **-BE**_2_ **) (eV)**
Vanadium	522.25	516.95	5.20
Manganese	654.77	643.46	11.31
Nickel	874.47	856.63	17.84

From the above results, it can be concluded the oxidation property of the mixed metal oxides used is not related to the redox properties of the transition metals used but could be a surface phenomenon.

### Thermogravimetric studies

**Figure 5:**
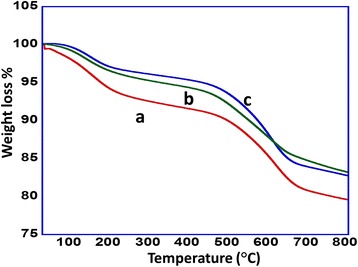
**TGA curves of the synthesized catalyst (a) V**_2_ **O**_5_ **(1%)-NiMnO, (b) V**_2_ **O**_5_ **(3%)-NiMnO, and (c) V**_2_ **O**_5_ **(5%)-NiMnO.**

The thermal stability of the as synthesized catalyst with different % loading of vanadium oxide nanoparticles were studied using TGA analysis. Temperature was programmed from 25°C to 800°C at a heating rate of 10°C min^−1^. It was observed that almost all the synthesized catalysts are thermally stable, yielding a maximum loss of weight of 20.23% at 800°C found in the case of 1% V_2_O_5_-nanoparticle-loaded catalyst making it to be the least thermally stable among the synthesized catalysts, while the catalysts with 3% V_2_O_5_ and 5% V_2_O_5_ can be assumed to be the most thermally stable catalysts with a least weight loss % of just 16.5% and 17.2%, respectively, at 800°C. A graphical illustration is given in Figure 5

.

### Evaluation of catalytic properties

#### Optimization of percentage of Vanadium oxide nanoparticles and calcination temperature

In order to ascertain the percentage composition of vanadium oxide nanoparticles to be supported on the nickel-manganese-mixed oxide for the best catalytic performance as an oxidation catalyst, a series of catalysts with varying percentages of vanadium oxide nanoparticles were synthesized and evaluated for their catalytic property, monitoring the oxidation of benzyl alcohol to benzaldehyde as a model reaction. The reaction was carried out at 100°C, while passing molecular O_2_ gas as a source of oxygen. During the study, a trend of steady increase in performance of the synthesized catalyst was observed with the increase in the composition percentage of vanadium oxide, which explains the influence of vanadium oxide nanoparticles on the catalytic performance. The catalyst with 1% and 3% vanadium oxide yielded 65.77% and 74.27% conversion product, respectively, within 75 min, while the catalyst with 5% vanadium oxide nanoparticles yielded 100% conversion product within the same time. In order to understand the effect of presence of vanadium oxide nanoparticles, a similar reaction was carried out in the presence of the catalyst without the vanadium oxide nanoparticles (i.e., NiMnO) which yielded a 52.56% conversion product. This indicated that vanadium oxide acts as a promoter for the selective catalytic oxidation.

**Figure 6:**
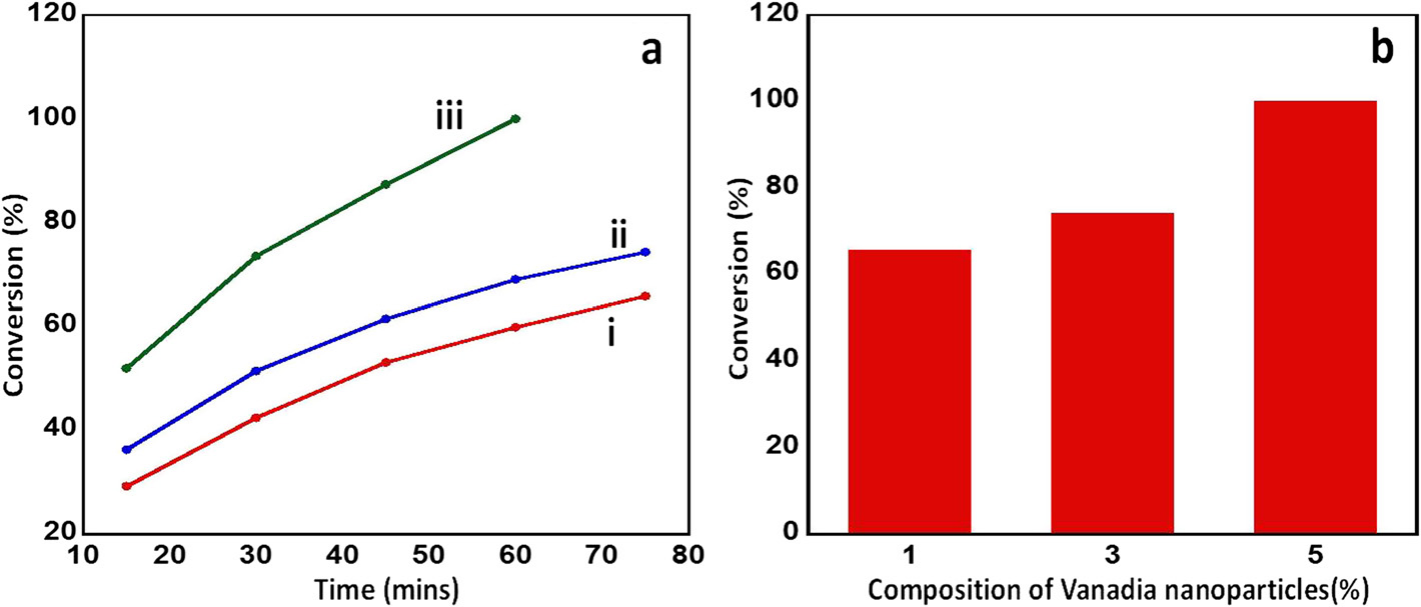
**Graphical illustration of the kinetics of the catalyst and bar chart depicting conversion product obtained.** Graphical illustration of the **(a)** kinetics of the catalyst for the conversion of benzyl alcohol to benzaldehyde using the synthesized catalyst (i) V_2_O_5_ (1%)-NiMnO, (ii) V_2_O_5_ (3%)-NiMnO, and (iii) V_2_O_5_ (5%)-NiMnO; **(b)** bar chart depicting conversion product obtained with different composition percentages of vanadia in the catalyst.

The kinetics of the reaction were studied by collecting the sample in regular intervals of 15 min and subjected to gas chromatography from which the percentage conversion was calculated. It was observed that the catalyst with 1% and 3% V_2_O_5_ start of by giving 30% and 37% conversion product, respectively, in the first 15 min of the reaction time. However, as the reaction proceeds, the rate slows down, and after 75 min, there was very slight change in the conversion product obtained; hence, the reaction was not carried on further. But the catalyst with 5% V_2_O_5_ yields about 52% conversion product in the first 15 min and gradually proceeds to the 100% conversion in 60 min. From this, it can be clearly stated that there is a promoter effect on the catalytic performance of the catalyst by incorporating vanadium oxide nanoparticles. The selectivity in all the above reactions was found to be >99%. A graphical illustration is given in Figure 6.

**Figure 7:**
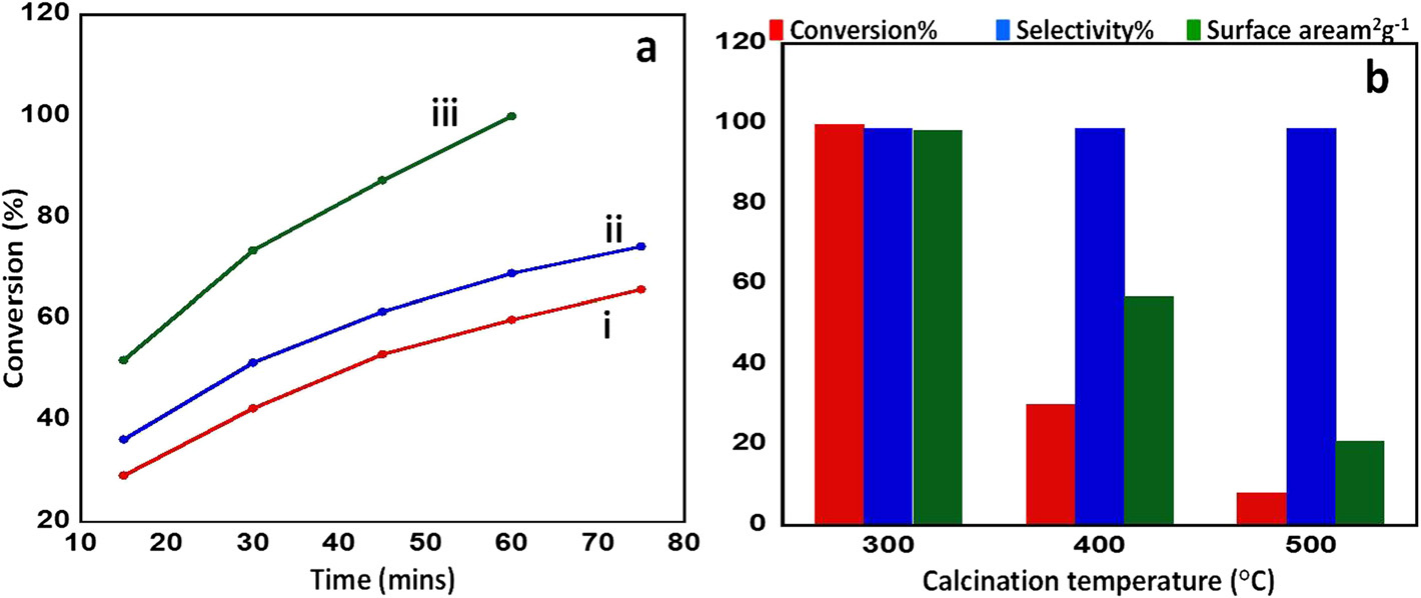
**Graphical illustration of the conversion of benzyl alcohol to benzaldehyde and effect of calcination temperature. (a)** Graphical illustration of the conversion of benzyl alcohol to benzaldehyde using the synthesized catalyst V_2_O_5_ (5%)-NiMnO calcined at different temperatures (i) 300°C, (ii) 400°C, and (iii) 500°C; **(b)** effect of calcination temperature on the catalytic properties.

Calcination temperature has an effect on the surface area and porosity, which in turn affects the catalytic performance as reported by Al-Fatesh and coworker [42]. In order to establish the above mentioned effect of calcination temperature on the synthesized catalyst, V_2_O_5_ (5%)-NiMnO catalyst, which yielded 100% conversion product in the earlier study, was taken and was calcined at different temperatures, i.e., 300°C, 400°C, and 500°C. Under similar reaction conditions, the conversion of benzyl alcohol to benzaldehyde was carried out. It was observed that with the catalyst V_2_O_5_ (5%)-NiMnO calcined at 300°C the reaction starts off by yielding a 51% conversion product in the first 15 min of the reaction time and yields a 100% conversion product within 60 min, while the catalyst calcined at 400°C and 500°C yielded 40% and 13% conversion product, respectively, in a reaction time of 180 min. The results are summarized in Table 3; a graphical illustration is given in Figure 7.

**Table 3:** **Effect of calcination temperature on the catalytic properties**

**Entry**	**Catalyst**	**Temperature (** **°C)**	**Conversion (%)**	**Selectivity (%)**
1	NiMnO	400	52.56	<99
2	V_2_O_5_ (5%)-NiMnO	300	100	<99
3	V_2_O_5_ (5%)-NiMnO	400	30	<99
4	V_2_O_5_ (5%)-NiMnO	500	8	<99

Reaction conditions: amount of catalyst 300 mg; reaction temperature 100°C; oxygen flow rate 20 mL min^−1^; benzyl alcohol 2 mmol; toluene 10 mL; reaction time 3 h.

### Optimization of source of oxygen

**Figure 8:**
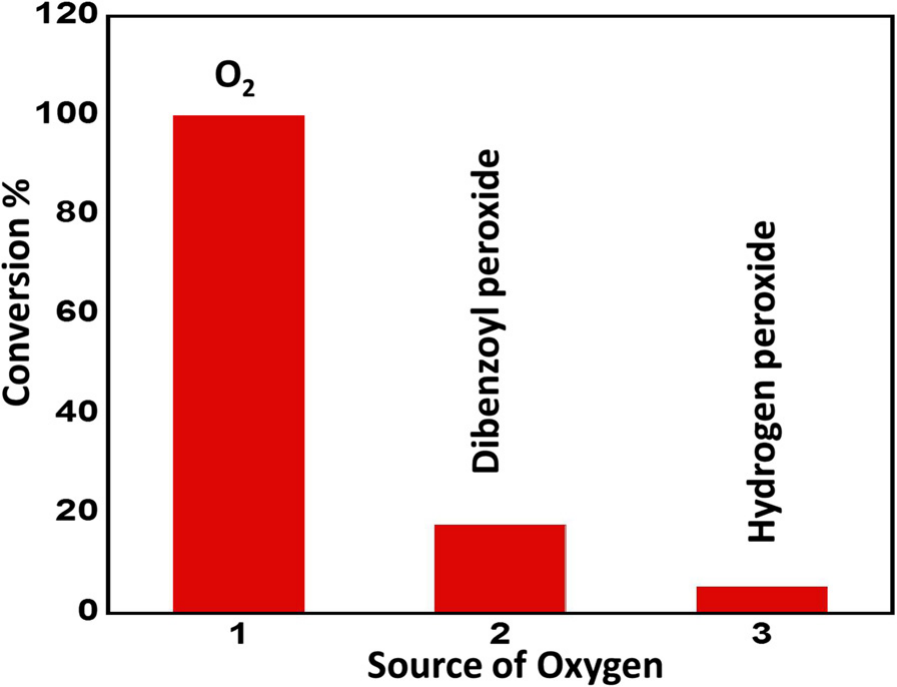
**Graphical illustration of the conversion of benzyl alcohol to benzaldehyde using the synthesized catalyst V**_2_ **O**_5_ **(5%)-NiMnO.** Graphical illustration of the conversion of benzyl alcohol to benzaldehyde using the synthesized catalyst V_2_O_5_ (5%)-NiMnO using different sources of oxygen.

The compatibility of the synthesized catalyst with different sources of oxygen such as dibenzoyl peroxide and hydrogen peroxide was tested. It was observed that there is a profound effect on the catalytic performance of the synthesized catalyst V_2_O_5_ (5%)-NiMnO calcined at 300°C when different sources of oxygen were employed. It was found that the catalyst displayed excellent performance with 100% conversion and >99% selectivity when molecular oxygen is used; while when dibenzoyl peroxide and hydrogen peroxide were used, the conversion product obtained was 17.75% and 5.31%, respectively. A graphical illustration is given in Figure 8. The results have been summarized in Table 4.

**Table 4:** **Effect of different sources of oxygen on the catalytic properties**

**Entry**	**Source**	**Conversion (%)**
1	O_2_	100
2	Dibenzoyl peroxide	17.75
3	Hydrogen peroxide	5.31

Reaction conditions: amount of catalyst 300 mg; reaction temperature 100°C; benzyl alcohol 2 mmol; toluene 10 mL; reaction time 2 h.

### Catalytic performance on different substrates of benzyl alcohol

From the conversion of benzyl alcohol to benzaldehyde which was used as a model reaction, it was ascertained that the best catalytic activity was displayed by V_2_O_5_ (5%)-NiMnO, calcined at 300°C, which was established by spectral studies to contain a mixture of cubic hexanickel manganese(IV) oxide and orthorhombic dinickel dioxide hydroxide. It can be concluded that the catalyst with a large surface area and the presence of orthorhombic dinickel dioxide hydroxide on the surface of the catalyst plays a crucial role. It was confirmed that the surface area plays a crucial role in the catalytic performance as the catalysts calcined at other temperatures too and were found to possess cubic hexanickel manganese(IV) oxide and orthorhombic dinickel dioxide hydroxide, but it did come out as the best catalyst among the synthesized catalysts which could be due to the low surface area. It was also confirmed that the catalyst performs best in the presence of molecular oxygen as a source of oxygen. In order to determine the catalytic performance of V_2_O_5_ (5%)-NiMnO (300°C), the reaction was carried out under a similar set of conditions using a series of substituted benzyl alcohols, containing 4-CH_3_, 4-OCH_3_, 4-Cl, 4-NO_2_, 4-C(CH_3_)_3_, 4-CF_3_, and 3-NO_2_ groups as different substrates, and their conversion to corresponding aldehydes was studied. It was found that the conversion product obtained was >60%, and selectivity displayed by the catalyst was >99%. It was observed that the catalyst selectively oxidizes aromatic alcohols, which was confirmed by the similar reaction carried out using citronellol as a substrate which yielded a conversion product of citronellal with 4%, unlike the results obtained from aromatic substrates. The results have been summarized in Table 5.

**Table 5:** **Selective oxidation of benzyl alcohol and derivatives into corresponding aldehydes in the presence of O**_2_ **as clean oxidant**

**R. No.**	**Reactants**	**Products**	**Conversion (%)**	**Selectivity**
1			100.00	>99
2			94.50	>99
3			89.36	>99
4			92.47	>99
5			100.00	>99
6			62.96	>99
7			59.32	>99
8			87.04	>99
9			4.07	>99

## Conclusions

We have synthesized vanadia-supported nickel manganese mixed oxide catalyst using facile sol–gel chemistry. Nanovanadia-supported nickel manganese oxide shows high activity and stability for the oxidation of benzyl alcohol using molecular oxygen as a source of oxygen. A synergistic effect between optimum calcination temperatures and the chemical kinetics of the reaction was observed, and it was confirmed that calcination temperature plays an important role forming an active and durable catalyst. It can be believed that this catalyst can be further used for the evaluation of its oxidative property for the synthesis of other important aromatic and aliphatic aldehydes.
